# Can magnetic resonance imaging (MRI) reduce lingual nerve injuries during mandibular third molar surgery? A scoping review

**DOI:** 10.1186/s12903-025-06589-9

**Published:** 2025-07-25

**Authors:** Rahmeh Alhyari, P. J. Ross, R. Sacco, A. AlHadidi, J. Mitchell, K. Khalaf, A. Lalli

**Affiliations:** 1 Institute of Dentistry, School of Medicine, Medical Sciences and Nutrition, University of Aberdeen, Foresterhill Campus, Cornhill Road, Aberdeen, AB25 2ZR UK; 2 Oral and Maxillofacial Surgery, Faculty of Dentistry, Zarqa University, P.O. BOX 2000, Zarqa, 13110 Jordan; 3 Aberdeen Biomedical Imaging Centre, School of Medicine, Medical Sciences and Nutrition, University of Aberdeen, Foresterhill Campus, Cornhill Road, Aberdeen, AB25 2ZR UK; 4 Faculty of Dentistry, Oral & Craniofacial Sciences, King’s College London, 29 Weston St, London, SE1 9SP UK; 5 College of Dentistry, New York University, 345 East 24th Street, NewYork, NY 10010 USA; 6 Oral and Maxillofacial Department, Aberdeen Royal Infirmary, NHS Grampian, Aberdeen, AB25 2ZN USA

**Keywords:** Lingual nerve, Preoperative imaging, Magnetic resonance imaging, Mandibular third molar surgery, Nerve injury

## Abstract

**Background:**

Recent advancements in MRI, with its superior soft tissue resolution and ionising radiation-free nature, provide a promising solution for the limitations of current imaging modalities. This review aims to evaluate whether MRI can be utilised to reduce the risk of lingual nerve (LN) injury during mandibular third molar surgery (M3M).

**Methods:**

Following PRISMA guidelines, the protocol was registered in PROSPERO (CRD42024625994). A systematic literature search was employed across MEDLINE/PubMed, Scopus, Web of Science, Cochrane Library, and Science Direct without language or date restrictions. Studies assessing MRI’s ability to visualise the LN and surgically relevant anatomy were included. The risk of bias was evaluated using ROBINS-I. Given the heterogeneity of included studies, this review was conducted as a scoping review to explore the range of evidence available, and findings were summarised through narrative synthesis.

**Results:**

Fourteen studies met the inclusion criteria. While none directly assessed whether MRI reduces the incidence of LN injury, it consistently demonstrated superior LN visualisation compared to conventional imaging. Among the sequences evaluated, Three-dimensional Double-Echo Steady-State with Water Excitation (3D-DESS-WE) and Sampling Perfection with Application-optimised Contrasts using different flip angle Evolutions with Short Tau Inversion Recovery (SPACE-STIR) provided nerve delineation and anatomical clarity. However, variability in scan duration, availability of MRI, the need for specialised image interpretation, and only moderate inter-observer agreement currently limit the clinical application of MRI in M3M surgery.

**Conclusion:**

Definitive evidence of the efficacy of MRI in reducing nerve injury during M3M is lacking. However, moderate to low quality evidence suggests that MRI offers superior visualisation of the LN compared to conventional imaging. Further clinical trials are needed to evaluate whether MRI’s enhanced preoperative visualisation translates into improved clinical outcomes from M3M surgery.

**Supplementary Information:**

The online version contains supplementary material available at 10.1186/s12903-025-06589-9.

## Background

Lingual nerve (LN) injury is a well-documented complication associated with mandibular third molar (M3M) surgery, with potential consequences including altered sensation in the tongue and neuropathic pain that can significantly impact a patient’s quality of life [1]. Reported injury rates for the LN range between 0.2 and 22% whereas the much more studied inferior alveolar nerve (IAN) injury rates range between 0.4 and 8.4% [1–3]. Preoperative visualisation and assessment of the IAN is permitted by conventional radiographic imaging modalities that show the bony mandibular canal outline, permitting better surgical planning and potentially reducing the risk of IAN injury [4, 5]. In contrast, the lingual nerve lacks a well-defined bony anatomical mark; therefore, it cannot be visualised with conventional imaging modalities such as panoramic radiography (OPG) and cone beam computed tomography (CBCT) [6], which may explain why preoperative strategies for lingual nerve risk assessment are not often evaluated in clinical practice [7].

LN injuries are fortuitously, often transient, with the majority resolving spontaneously within months, such that permanent deficits occur in only 0.1–2% of cases [8, 9]. Despite the overall low incidence of permanent injury, the risk of significant sensory impairment justifies ongoing efforts to reduce these life-changing injuries following elective surgery. Intraoperative precautions, such as avoiding the reflection of lingual flaps, limiting excessive force during tooth elevation, and maintaining an atraumatic surgical approach, have been shown to minimise but not eliminate the risk of LN injury [10]. Consequently, LN injuries remain prevalent as up to 60% of the population will require M3M removal in their lifetime [11]. Therefore, preoperative visualisation of the LN remains a critical gap in the current imaging-based risk assessment for M3M surgery.

Recent advancements in Magnetic Resonance Imaging (MRI), with its superior soft tissue resolution and ionising radiation-free nature, provide a promising solution for the preoperative assessment of LN injury risk [12]. Studies have demonstrated MRI’s capability in providing detailed anatomical visualisation of the LN and potential anatomical variations [13]. Given the significant clinical implications of LN injury and MRI’s potential to overcome the limitations associated with conventional imaging, this review aims to evaluate whether MRI can be utilised to reduce the risk of LN injury during M3M surgery.

## Methodology

### Study design and ethical considerations

Due to the variability in outcomes, imaging parameters, and lack of clinical trials, this review adopts a scoping review methodology to comprehensively map the current evidence base. It was conducted following PRISMA for Scoping Reviews (PRISMA-ScR) guidelines [14]. The protocol was prospectively registered in the PROSPERO database (CRD42024625994).

### Research question and eligibility criteria

This review was designed to assess the role of MRI in reducing the risk of nerve injury during M3M surgery. The eligibility criteria were structured using the PICOS framework and summarised in Table 1. Only human studies involving patients or healthy volunteers undergoing MRI to visualise the third molar region were included. Exclusion criteria covered non-original research (review articles, editorials, case reports, letters), studies involving cadaveric specimens, phantom models, or animal subjects, and studies using only contrast-enhanced MRI due to their limited applicability in dental practice and clinical feasibility concerns.

**Table 1:** PICOS framework and eligibility criteria

Criterion	Details
Population (P)	Human patients or healthy volunteers undergoing MRI of the MTM region.
Intervention (I)	MRI showing the mandible.
Comparator (C)	Standard imaging modalities or no imaging.
Outcomes (O)	Nerve injury incidence, nerve visualisation
Study design (S)	Randomised controlled trials, cohort studies, case-control studies, and case series.

### Data sources and search strategies

A comprehensive literature search was performed across multi-electronic databases: MEDLINE/PubMed (https://pubmed.ncbi.nlm.nih.gov/), Scopus (https://www.scopus.com/), Web of Science (https://www.webofscience.com/), Cochrane Library (https://www.cochranelibrary.com/) and Science Direct (https://www.sciencedirect.com/). Additional searches were conducted in grey literature sources, including conference proceedings and clinical trial registries, to minimise selection and publication bias. No restrictions were applied to language or publication date. The last search was conducted in January 2025.

The search strategy (Table 2) was designed to systematically identify studies considering MRI to visualise the LN or MTM area. Three primary search components were used: imaging techniques, third molar-related, and nerve-related terms. Boolean operators (AND/OR) were applied to combine these components into a structured search. The search strategy consisted of two phases; the initial strategy aimed to identify studies directly evaluating MRI’s role in reducing LN injury risk. Since no studies met this criterion, a secondary search strategy was employed to identify research on MRI’s ability to visualise relevant anatomical structures to assess its potential contribution to risk assessment and surgical planning.

### Study selection, data extraction, and management

The studies identified in each database were exported to EndNote reference management software (Clarivate, Philadelphia, USA) for storing, organising, and deduplicating retrieved citations before the screening process. Rayyan AI (Rayyan Systems Inc., Doha, Qatar) was used as a web-based platform to facilitate blinded title and abstract screening by two independent reviewers. Studies without an available abstract were retained for the next selection phase. Articles meeting the inclusion criteria underwent further full-text evaluation. Any disagreements were resolved through discussion between the two reviewers, and if an agreement could not be reached, the final decision would be made by a third independent reviewer.

A structured data extraction form was developed using Microsoft Excel. The extracted parameters encompassed five main categories. Study identification included details such as study ID, author(s), title, year of publication, journal, and country. Study characteristics covered the study design, ethical approval status, duration, and stated objectives. Eligibility criteria described the inclusion and exclusion criteria used for participant selection for each study. Imaging details and analysis involved information on the MRI vendor, field strength, coil type, MRI sequences and protocols, contrast administration, total scan duration, comparator imaging modalities (if any), image analysis methods, and the number and expertise of image reviewers. Lastly, results and outcomes included participant demographics, statistical findings, observed anatomical structures, and the methods used for outcome evaluation. Given the heterogeneity of study designs, populations, and outcomes, a narrative synthesis approach was adopted. No Missing data were addressed.

### Risk of bias assessment

The risk of bias in the included studies was assessed independently by two reviewers using the Risk of Bias in Non-randomised Studies of Interventions (ROBINS-I) tool [15]. Any disagreements between reviewers were resolved through discussion or consultation with a third reviewer.

ROBINS-I was selected over QUADAS-2 due to the clinical rather than purely diagnostic accuracy focus of this review [15, 16]. While QUADAS-2 typically requires a universally accepted reference standard to assess diagnostic accuracy, such a standard does not yet exist for lingual nerve visualisation. Moreover, ROBINS-I effectively addresses key methodological issues inherent in observational studies.

## Results

### Study selection and characteristics

**Fig. 1:**
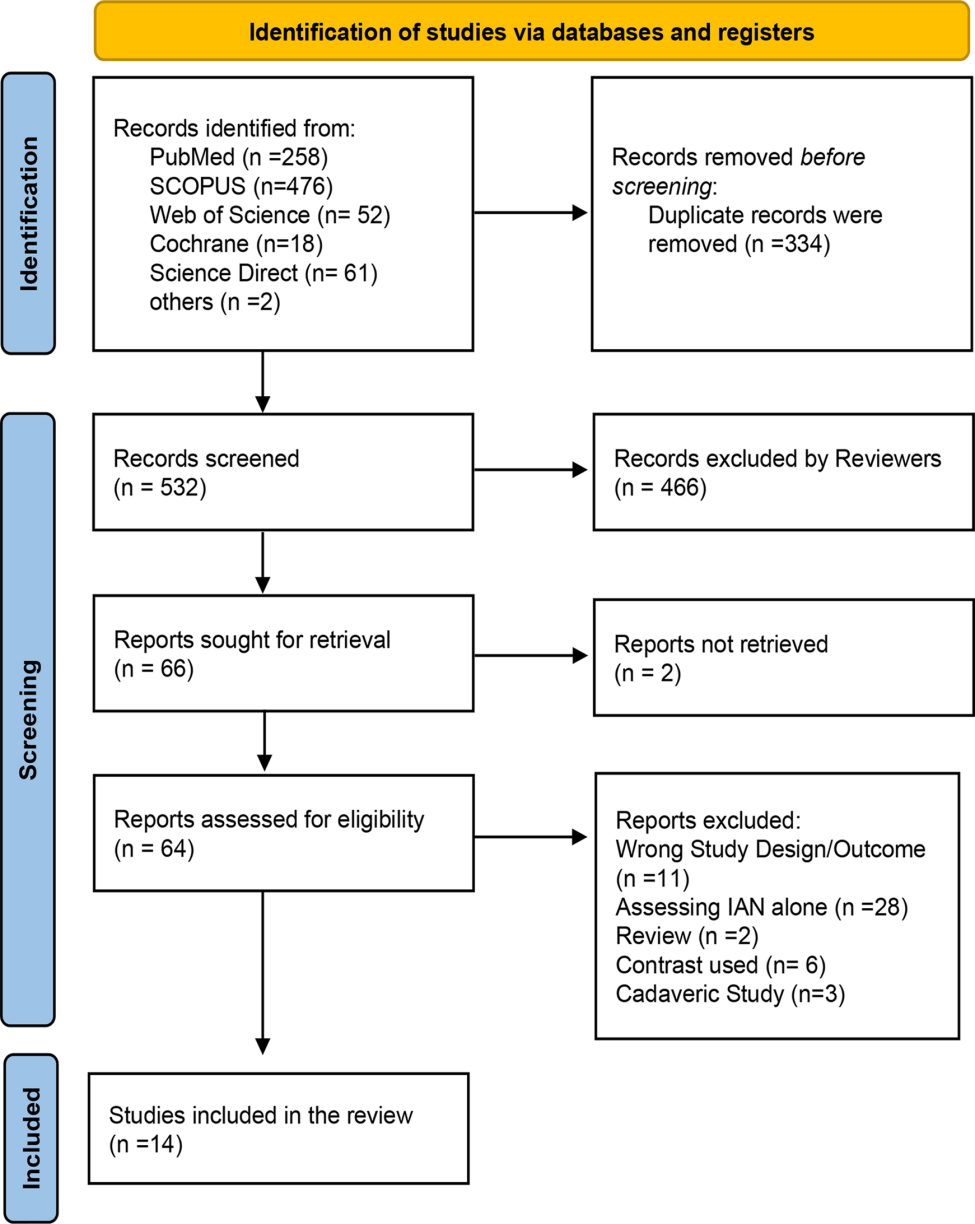
PRISMA flowchart showing study selection process

A total of fourteen studies met the eligibility criteria. Four studies studied LN imaging alone, while the remaining ten evaluated both LN and IAN together. None were randomised controlled trials or large comparative cohorts examining direct nerve injury outcomes; most included studies were observational or feasibility imaging studies. The PRISMA diagram (Fig. 1) outlines the screening and selection followed. The characteristics of the included studies, including study design, sample size and characteristics, are summarised in Table 3, while the MRI protocols and observed structures are detailed in Table 4.

**Fig. 2:**
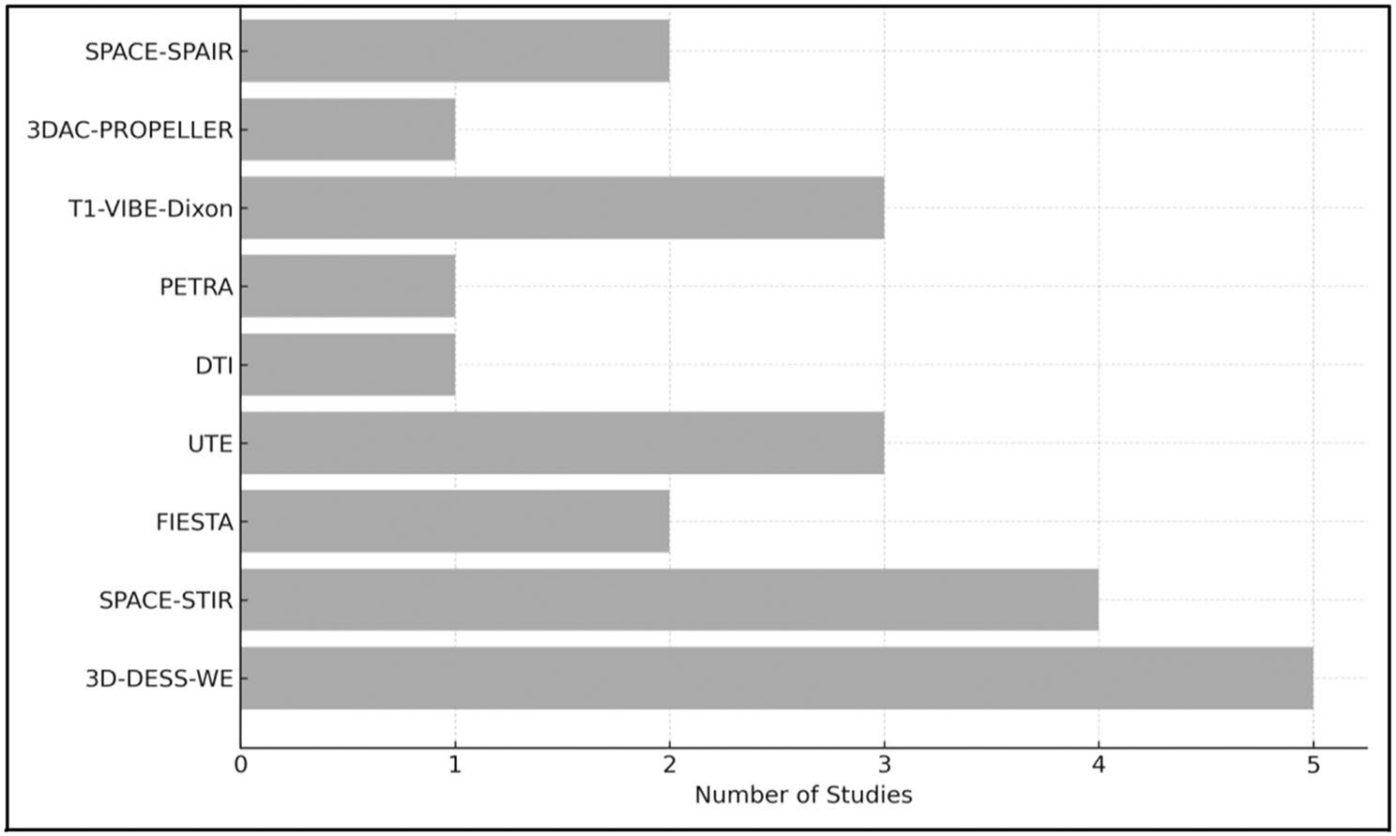
The distribution of MRI sequences used across the 14 included studies

The included studies span a publication period from 1997 to 2024, with an increasing publication trend over recent years. Sample sizes ranged from as low as 6 up to 86 participants. Overall, among the studies that reported age data, participant ages ranged from 18 to 84 years, although several studies did not provide precise age ranges or means. Regarding MRI parameters, Field strengths typically were 3.0 Tesla in newer studies, whereas older publications, such as Miloro et al. 1997, used a 1.5T scanner. The MRI sequences used across studies are presented in Table 4. A comparative Table 5 summarises the reported strengths and limitations of MRI sequences. To provide a clearer visual representation of the sequence usage trends, Fig. 2 displays the frequency of each MRI sequence employed across the 14 included studies. Notably, 3D-DESS-WE was the most commonly applied.

### MRI sequences that offer the best visualisation of nerves

Across the included studies, MRI consistently demonstrated a high capacity in visualising both the LN and IAN, particularly when using higher magnetic field strengths (3.0 Tesla). Among the MRI sequences evaluated, 3D-DESS-WE and SPACE-STIR were most often reported as the most clinically practical and diagnostically beneficial sequences. The 3D-DESS-WE sequence offers excellent continuous nerve visualisation, reproducibility, and balanced spatial resolution with shorter scan times, making it particularly suitable for dental applications [6, 17, 18]. The SPACE-STIR sequence provides high soft-tissue contrast, superior nerve-to-muscle differentiation, and more comprehensive anatomical detail, although with slightly longer acquisition times [6].

Jiang et al. (2024) [19] and Al-Haj Husain et al. (2023) [20] reported an effective mapping of the LN pathway. Mazza et al. (2020) also showed that MRI enables clear visualisation of the lingual nerve in the region of the M3M and from its origin to the mylohyoid muscle [21]. Whereas, Fujii et al. (2015) quantified LN visualisation with an average clarity score of 3.80 out of 4 [22]. Approximate MRI scan times varied, with single-sequence protocols such as 3D-DESS-WE and FIESTA typically lasting 8–15 min, and multi-sequence protocols often extending to 15–25 min.

### MRI capabilities compared to CBCT

This review confirms that MRI offers good LN visualisation [19, 20, 22]. Additionally, MRI has the ability to detect IAN anatomical variations and accessory nerve branches, which may not be reliably visualised with conventional imaging. For example, Beck et al. (2021) noted that MRI was superior in identifying accessory IAN branches compared to CBCT, potentially influencing surgical planning [23]. However, these benefits remain theoretical, as MRI’s clinical utility is yet to be proven definitively [24, 25].

### Overall findings and feasibility of meta-analysis

Due to substantial clinical and methodological heterogeneity, including varied MRI protocols, field strengths, participant characteristics, and absence of unified clinical endpoints, a meta-analysis was not feasible. Across the included studies where MRI was compared to other imaging modalities, MRI consistently demonstrated superior capability in visualising neural structures relevant to M3M surgery.

### Risk of bias assessment

**Fig. 3:**
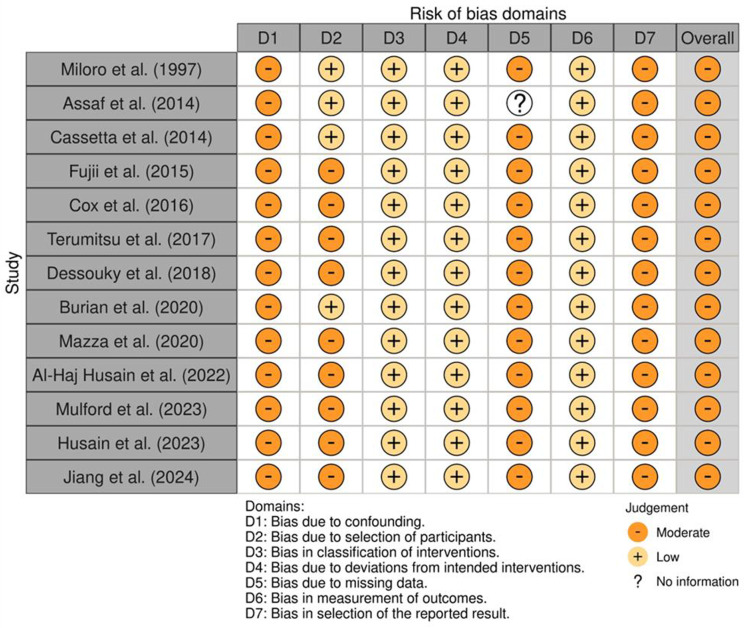
ROBINS-I “traffic light” plot of risk of bias across included studies

The risk of bias for each included study was evaluated using the Risk of Bias in Non-randomised Studies of Interventions (ROBINS-I) tool. Assessments across the seven ROBINS-I domains are summarised visually in Fig. 3, generated using the Risk of Bias Visualisation (robvis) tool [26]. Overall, the studies demonstrated a moderate risk of bias, highlighting the necessity for larger, high-quality prospective cohorts or randomised controlled trials to produce more definitive clinical conclusions. The most common concerns identified were confounding bias, bias arising from participant selection, and bias related to missing outcome data. Addressing these biases through robust study designs would significantly enhance the strength and reliability of future evidence.

## Discussion

This review, conducted according to multiple-reviewer quality assessment standards, identified only studies corresponding to Levels 2b and 3 of the Oxford Centre for Evidence-Based Medicine hierarchy [27]. Despite the overall moderate quality of included studies, MRI demonstrates excellent anatomical visualisation of the lingual and inferior alveolar nerves. However, none of the included studies provided direct clinical evidence linking MRI-guided surgical planning to a reduction in nerve injury. The current literature lacks randomised controlled trials or longitudinal outcome studies that evaluate whether superior imaging translates into improved surgical safety or patient outcomes.

This review supports MRI’s potential role in surgical planning for M3M surgery. MRI’s non-ionising imaging properties and excellent soft-tissue contrast, permitting identification of all clinically relevant structures, including the LN, IAN, M3M, and lingual plate, which suggests it could potentially be superior to conventional pre-operative imaging modalities in M3M surgery. While previous systematic reviews (Al-Haj Husain et al., 2021 [28] and Al-Haj Husain et al., 2023 [29]) have effectively explored MRI’s diagnostic capabilities, they primarily focused on comparative visualisation and technical feasibility and stopped short of exploring MRI’s impact on surgical outcomes. This review explores that clinically important gap by evaluating MRI’s efficacy in the preoperative context, directly linking its diagnostic features with potential clinical impact and specifically reduction of nerve injuries.

Enhanced visualisation of the LN could significantly improve surgical planning and decision-making by providing surgeons with detailed preoperative images. The resultant increased anatomical understanding allows for more informed choices about surgical approaches, such as partial or total M3M removal, as well as the intraoperative technique, such as where to place incisions, section teeth, or remove bone. All of which could potentially reduce the incidence of iatrogenic nerve injuries.

### MRI offers better visualisation compared to conventional modalities

Conventional radiographic methods, including OPG and CBCT, primarily visualise bony structures and provide only indirect inference of IAN position whilst offering minimal or no anatomical information on the LN [18, 25, 30]. Either nerve, when damaged during surgery, could significantly affect a patient’s quality of life, causing sensory disturbances, pain, and functional impairment, yet the clinical literature predominantly focuses only on the IAN due to the relative ease of visualisation [31, 32]. Consequently, preventing iatrogenic damage to the LN, although equally clinically important, is relatively ignored.

### Clinical constraints of MRI in M3M surgery

Despite MRI’s promising capabilities, several limitations were reported. Longer acquisition times associated with certain MRI techniques, such as MR Neurography protocols using T2-weighting and specific sequences such as STIR, could be problematic for patients and healthcare service providers [33, 34]. MRI scan durations varied considerably, ranging from short single-sequence protocols (8–12 min) to comprehensive multi-sequence protocols lasting 25 min or longer [22, 35]. It appears that shorter MRI protocols, such as single-sequence 3D-DESS-WE, offer practical clinical applicability and improved patient compliance [17]. In contrast, multi-sequence protocols, often extending beyond 15 min, provided richer anatomical detail but presented greater challenges such as patient discomfort, motion artefacts, and higher costs [34, 36].

Another critical limitation relates to metal-induced artefacts and safety considerations [37]. Artefact severity in MRI increases linearly with magnetic field strength, resulting in larger signal voids at 3.0T compared to 1.5T. Furthermore, metallic implants within the imaging field can pose significant safety hazards, including device movement and radiofrequency-induced heating, imposing strict consideration of MRI safety or conditionality. While both MRI and CBCT are susceptible to metal artefacts, the nature and severity differ, and comparative data remain limited within the literature. Importantly, given the increasing prevalence of dental implants and restorations in the adult population, patient eligibility for MRI-based assessment could be restricted.

Additionally, the broader feasibility of MRI integration into M3M surgical planning warrants consideration. Unlike X-ray and CBCT, which are widely available and relatively inexpensive, MRI systems require substantial infrastructure investment and operating costs, potentially limiting their adoption in financially constrained healthcare systems unless significant benefits can be determined.

Moreover, variability in image interpretation among observers and only moderate inter-observer agreement suggest that surgeons could require additional training to usefully interpret MR imaging. A standardised training protocol for utilising MRI images is crucial to enable broader acceptability within dental practice, enhance accurate interpretation, and facilitate safer and more informed surgical decision-making.

### Study limitations and directions for future research

A major limitation of this review is the absence of randomised controlled trials or robust, high-quality prospective studies directly comparing MRI with conventional imaging modalities using defined clinical outcomes, such as postoperative nerve injury incidence. Although MRI has a potentially superior anatomical representation compared to conventional imaging, no study in this review attempted to correlate this with clinical outcomes in M3M surgery. This evidence gap significantly limits the clinical applicability and generalisability of current findings. Interestingly, CBCT, although now well established as a gold standard imaging modality in high-risk M3M surgery, also lacks conclusive evidence for its role in reducing nerve injury [24, 25].

Thus, MRI has considerable promise in M3M surgery, but high-quality research is needed with key outcomes such as the incidence and severity of postoperative sensory disturbances, patient-reported outcomes, surgical complication rates and cost-effectiveness analyses. Other areas for research include standardisation of MRI protocols, especially adopting short-duration sequences, to enhance clinical relevance and comparability across research studies.

The emergence of ddMRI systems, operating at approximately 0.55T, offers potentially adequate image quality while significantly reducing operational costs and infrastructure demands compared to conventional high-field MRI [38, 39] The use of ddMRI in M3M surgery is yet to be reported as are other novel low-energy MR technologies such as fast field cycling MRI (FFC-MRI) [40].

## Conclusion

This scoping review provides an overview of MRI in preoperative assessment for M3M surgery. While MRI demonstrates superior anatomical visualisation compared to conventional imaging modalities, especially with sequences such as 3D-DESS-WE and SPACE-STIR, no included studies directly evaluated its impact on reducing the incidence of nerve injuries. The heterogeneity of imaging protocols, outcome measures, and study designs precluded meta-analysis and limited direct clinical applicability.

Future research should prioritise the standardisation of imaging protocols and prospective clinical trials that assess whether MRI-based preoperative planning can reduce nerve injury rates.

**Table 2:** Search strategies used in each database and their number of entries

Database	Search strategy
PubMed	(((“MRI“[Title] OR “Magnetic Resonance Imaging“[Title] OR “Nuclear Magnetic Resonance Imaging“[Title] OR “diffusion tensor imaging“[Title] OR “DTI“[Title] OR “short tau inversion recovery“[Title])) AND (((“wisdom tooth“[Title/Abstract] OR “wisdom teeth“[Title/Abstract] OR “third molar“[Title/Abstract] OR “mandibular third molar“[Title/Abstract] OR “lower third molar“[Title/Abstract] OR “M3M“[Title/Abstract] OR “Trigeminal nerve“[Title/Abstract] OR “Mandibular nerve“[Title/Abstract] OR “Inferior alveolar nerve“[Title/Abstract] OR “Inferior dental nerve“[Title/Abstract] OR “Lingual nerve“[Title/Abstract])))
SCOPUS	(TITLE (“MRI” OR “Magnetic Resonance Imaging” OR “Nuclear Magnetic Resonance Imaging” OR “diffusion tensor imaging” OR “DTI” OR “short tau inversion recovery”) AND (TITLE-ABS-KEY (“wisdom tooth” OR “wisdom teeth” OR “third molar” OR “mandibular third molar” OR “lower third molar” OR “M3M” OR “Trigeminal nerve” OR “Mandibular nerve” OR “Inferior alveolar nerve” OR “Inferior dental nerve” OR “Lingual nerve”)))
WEB OF SCIENCE	(TI=((“MRI” OR “Magnetic Resonance Imaging” OR “Nuclear Magnetic Resonance Imaging” OR “diffusion tensor imaging” OR “DTI” OR “short tau inversion recovery”))) AND AB=((“wisdom tooth” OR “wisdom teeth” OR “third molar” OR “mandibular third molar” OR “lower third molar” OR “M3M” OR “Mandibular nerve” OR “Inferior alveolar nerve” OR “Inferior dental nerve” OR “Lingual nerve”))
COCHRANE	(“magnetic resonance imaging” OR MRI OR “nuclear magnetic resonance imaging” OR NMR OR “diffusion tensor imaging” OR DTI OR “ultrashort echo time” OR UTE) AND (“inferior alveolar nerve” OR “lingual nerve” OR “mandibular nerve” OR “mandibular third molar” OR “wisdom tooth” OR “third molar”) in Title Abstract Keyword - (Word variations have been searched)
SCIENCE DIRECT	Title, abstract, keywords: ((“MRI” OR “Magnetic Resonance Imaging”) AND (“inferior alveolar nerve” OR “lingual nerve” OR “mandibular nerve” OR “mandibular third molar” OR “wisdom tooth” OR “third molar”))

**Table 3:** Summary of included studies, target populations and study characteristics

Study	Study Design	Sample size and characteristics
Miloro et al. [41] (1997)	Prospective	10 healthy volunteers (20 LNs)
Assaf et al. [42] (2014)	Prospective	12 healthy volunteers
Cassetta et al. [43] (2014)	Retrospective	78 patients underwent head and neck MRI
Fujii et al. [22] (2015)	Retrospective	85 patients underwent MRI for salivary gland lesion evaluation.
Cox et al. [34] (2016)	Retrospective	17 patients with suspected PTN. 12 with iatrogenic LN/IAN trauma.
Terumitsu et al. [44] (2017)	Prospective	19 patients with unilateral LN/IAN injuries.
Dessouky et al. [33] (2018)	Retrospective	42 patients (24 with clinically suspected PTN after MTM surgery and 18 controls)
Burian et al. [6] (2020)	Prospective	30 healthy volunteers
Mazza et al. [21] (2020)	Prospective	24 patients undergoing MRI for unrelated medical reasons
AlHaj Husain et al. [17] (2022)	Prospective	19 patients (38 LNs) with an indication for MTM surgery
Al-Haj Husain et al. [20] (2023)	Prospective	21 patients clinically asymptomatic for head and neck pathologies
Mulford et al. [36] (2023)	Prospective	6 healthy volunteers
Husain et al. [35] (2024)	Prospective	11 patients undergoing MTM surgery
Jiang et al. [19] (2024)	Prospective	25 patients undergoing MTM surgery (50 LNs)

Abbreviations: PTN = Peripheral Trigeminal Neuropathy; TN = Trigeminal Nerve

**Table 4:** Summary of MRI field strengths, MRI sequences and reported visualised structures in the included studies

Study	Field Strength	MRI Sequence	Significant Quantitative Result	Structures Visualised
Miloro et al. [41] (1997)	1.5T	PETRA	In the M3M region, only 10% of LN was above the lingual crest, and 25% was in direct contact with the plate.	LN, MTM, LP
Assaf et al. [42] (2014)	3.0T	T1, T2, STIR “black bone”	Evaluation of the visibility of the LN was nearly as good as the delimitation of IAN (mean score 1.75 +/- 0.5)	LN, IAN, MTM, dental pulp
Cassetta et al. [43] (2014)	3.0T	3D-FIESTA, T1 fast SPGR	N/A	LN, IAN, TN branches
Fujii et al. [22] (2015)	3.0T	3D-DESS-WE	The detection of the LN and IAN with excellent average visibility scores of 3.80 and 3.99, respectively.	LN, IAN, TN branches
Cox et al. [34] (2016)	1.5T	T2 SPAIR, T1 SE, CISS 3D, DTI, STIR SPACE, DWI-PSIF	The abnormalities were seen in the IAN (13/17), LN (2/17), and both IAN and LN (2/17).	LN, IAN, NVB
Terumitsu et al. [44] (2017)	3.0T	3DAC-PROPELLER DWI	N/A	LN, IAN, Neuroma
Dessouky et al. [33] (2018)	1.5T, 3.0T	SPAIR, CISS, DTI, STIR SPACE, PSIF, BFFE, 3T SHINKEI	The area under the curve revealed an accuracy of 0.83–0.84 for IAN, and 0.77–0.78 for LN.	LN, IAN
Burian et al. [6] (2020)	3.0T	3D STIR, 3D-DESS-WE, 3D T1 FFE	Image quality was rated as excellent in 95% and as good in 5%. 100% detected LN and IAN.	LN, IAN
Mazza et al. [21] (2020)	3.0T	SSFP/FIESTA	The image quality at the level of the third molar site, on axial plane is good in the 83,3% of cases, and blurred in only one case (4,2%) and on a frontal plane is good in the 75% and blurred in the 8,3%.	LN
AlHaj Husain et al. [17] (2022)	3.0T	3D-DESS-WE	The average LN continuity score was 3.3 ± 0.46 out of 5.	LN
Al-Haj Husain et al. [20] (2023)	3.0T	3D-DESS-WE, SPACE-STIR, SPACE-SPAIR, VIBE-Dixon, UTE	Average image quality was good (3.29 ± 0.83) for all MRI protocols, with UTE providing the best image quality (3.52 ± 0.62) and no to minor artefacts (2.56 ± 0.6).	IAN, LN, Dental pulp, MC, TMJ, Teeth
Mulford et al. [36] (2023)	3.0T	DTI, T2-STIR-SPACE	Tracts in IAN exhibited the strongest signal (58%), compared to a lower signal of LN (17%).	LN, IAN
Husain et al. [35] (2024)	3.0T	UTE, 3D-DESS-WE, SPACE-STIR, T1-VIBE-Dixon	The mean score of full diagnostic interpretability, for UTE (3.64 ± 0.498) and good for VIBE (3.09 ± 1.036), while it was lowest for SPACE STIR (2.837 ± 1.049). Nerve continuity analysis yielded excellent results, with the entire course of the inferior alveolar nerve is consistently visible in all cases for DESS and SPACE STIR (both 4 ± 0).	IAN, LN, TN branches
Jiang et al. [19] (2024)	3.0T	3D T2 FFE/FRACTURE fusion	The fusion images demonstrated that the LN continuity score was good (3.00), with 88% of LNs displayed continuously at the MTM level. Intra-reader agreement for nerve continuity was moderate (κ = 0.527).	LN, LP

Abbreviations: T = Tesla, Lingual Plate = LP, TN = Trigeminal nerve; MRN = Magnetic Resonance Neurography; NVB = NeuroVascular Bundle; MC = Mandibular Canal; TMJ = Temporomandibular joint; T1 = Longitudinal Relaxation Time, T2 = Transverse Relaxation Time, PETRA = Pointwise Encoding Time Reduction with Radial Acquisition; STIR = Short Tau Inversion Recovery; 3D-FIESTA = 3D Fast Imaging Employing Steady-state Acquisition; SPGR = Spoiled Gradient-Recalled Echo; 3D-DESS-WE = 3D Double-Echo Steady-State with Water Excitation; SPAIR = Spectral Attenuated Inversion Recovery; SE = Spin Echo; CISS = Constructive Interference in Steady State; DTI = Diffusion Tensor Imaging; PSIF = Reversed Fast Imaging with Steady-State Precession; DWI-PSIF = Diffusion-Weighted Imaging with PSIF sequence; PROPELLER = Periodically Rotating Overlapping Parallel Lines with enhanced reconstruction; 3DAC-PROPELLER DWI = 3D Anisotropy Contrast - PROPELLER acquisition with Diffusion Weighted Imaging; BFFE = Balanced Fast Field Echo; SHINKEI = Sheath Inked Rapid Acquisition with Refocused Echoes; FFE = Fast Field Echo; SSFP = Steady-State Free Precession; SPACE = Sampling Perfection with Application-optimised Contrasts using different flip angle Evolutions; VIBE = Volumetric Interpolated Breath-hold Examination; UTE = Ultrashort Echo Time; FRACTURE = Fast field echo resembling a CT using restricted echo-spacing

**Table 5:** Comprehensive MRI sequence comparative analysis

MRI Sequence	Advantages	Limitations
3D DESS WE	High reproducibility, excellent for continuous nerve visualisation and surgical planning [6, 17, 22].	Distal LN is occasionally less visible, with moderate inter-reader variability [6, 22].
3DAC-PROPELLER	Excellent depiction of nerve integrity post-trauma; good correlation to sensory disturbances [44].	Restricted clinical utility primarily for trauma-related nerve changes [44].
3D T2 FFE & FRACTURE fusion	Reliable continuous visualisation of LN; suitable for measuring cortical bone thickness around LN [19].	Artefacts affecting image clarity, moderate reader variability [19].
UTE	Excellent for visualising positional relationships, teeth and periapical regions [20]. Can be provided as panoramic radiograph-like images. Exhibit no or minor artefacts [18, 20].	Reduced visibility in distal lingual nerve segments, incomplete continuous visualisation in a significant proportion of cases, affected by reader variability and anatomical factors [18, 20].
FIESTA	Rapid scanning, high SNR, High spatial resolution, clear LN visualisation proximal to MTM [21, 45].	Reduced clarity and nerve visualisation in the distal region, and requires expert radiologist interpretation [21, 45].
PETRA	Precise anatomical localisation of LN [41].	Older technology: limited spatial resolution compared to newer sequences [41].
SPACE-STIR/SPACE-SPAIR/T1-VIBE-Dixon	Good overall image quality and nerve continuity visualisation. Excellent nerve-muscle contrast, superior SNR compared to conventional sequences [6, 20, 46].	Longer acquisition times, Motion artefacts and metallic artefacts can affect diagnostic clarity, dedicated interpretation training, and are not consistently validated [6, 20, 46].

Abbreviations: 3D-DESS-WE = Double-Echo Steady-State with Water Excitation; DTI = Diffusion Tensor Imaging; 3DAC-PROPELLER = 3D Anisotropy Contrast with Periodically Rotating Overlapping Parallel Lines with enhanced reconstruction acquisition; 3D T2-FFE = 3D T2-weighted Fast Field Echo; FRACTURE = Fast field echo resembling a CT using restricted echo-spacing; SSFP = Steady-State Free Precession; FIESTA = Fast Imaging Employing Steady-State Acquisition; PETRA = Pointwise Encoding Time Reduction with Radial Acquisition; SPACE = Sampling Perfection with Application-optimised Contrasts using different flip angle Evolutions; STIR = Short Tau Inversion Recovery; SPAIR = Spectral Attenuated Inversion Recovery; VIBE = Volumetric Interpolated Breath-hold Examination; UTE = Ultrashort Echo Time; SNR = Signal-to-noise ratio

## Electronic supplementary material

Below is the link to the electronic supplementary material.

Supplementary Material 1

## Data Availability

The datasets used during the current study are available from the corresponding author upon reasonable request.

## Declarations

### Author information

RA is a PhD candidate in Dentistry with a Dentomaxillofacial Radiology interest at the University of Aberdeen. AL is RA’s academic supervisor, an Oral Surgery Consultant, an academic Senior Clinical Lecturer and the Postgraduate Research Program Coordinator at the University of Aberdeen. KK is a Professor of Orthodontics and Director of the Institute of Dentistry at the University of Aberdeen. JR is a PI in the Medical Imaging Technologies research theme, with a focus on MRI, at the University of Aberdeen. AA is a Clinical Associate Professor of Oral and Maxillofacial Radiology at New York University, USA. RS is an Oral Surgery Specialist and Clinical Lecturer at King’s College London, UK. JM is a clinician and researcher with expertise in Oral Surgery at NHS Grampian, UK. The team combines experienced academics, clinicians and researchers in oral surgery and radiology, contributing multidisciplinary expertise to this review.

## Abbreviations

MRI: Magnetic Resonance Imaging
LN: Lingual Nerve
M3M: Mandibular Third Molar
OPG: Orthopantomogram
CBCT: Cone Beam Computed Tomography
IAN: Inferior Alveolar Nerve
PRISMA: Preferred Reporting Items for Systematic Reviews and Meta-Analyses
PRISMA-ScR: PRISMA Extension for Scoping Reviews
PROSPERO: International Prospective Register of Systematic Reviews
PICOS: Population, Intervention, Comparator, Outcomes, Study Design
ROBINS-I: Risk Of Bias In Non-randomised Studies - of Interventions
DTI: Diffusion Tensor Imaging
STIR: Short Tau Inversion Recovery
SPACE-STIR: Sampling Perfection with Application-optimised Contrasts using different flip angle Evolutions with Short Tau Inversion Recovery
SPACE-SPAIR: Sampling Perfection with Application-optimised Contrasts using different flip angle Evolutions with Spectral Attenuated Inversion Recovery
SPAIR: Spectral Attenuated Inversion Recovery
CISS: Constructive Interference in Steady State
SE: Spin Echo
PSIF: Reversed Fast Imaging with Steady-State Precession
DWI-PSIF: Diffusion-Weighted Imaging with PSIF sequence
PROPELLER: Periodically Rotating Overlapping Parallel Lines with Enhanced Reconstruction
3DAC-PROPELLER: 3D Anisotropy Contrast - PROPELLER Acquisition with Diffusion Weighted Imaging
BFFE: Balanced Fast Field Echo
SHINKEI: Sheath Inked Rapid Acquisition with Refocused Echoes
FFE: Fast Field Echo
SSFP: Steady-State Free Precession
3D-FIESTA: 3D Fast Imaging Employing Steady-state Acquisition
3D-DESS-WE: 3D Double-Echo Steady-State with Water Excitation
VIBE: Volumetric Interpolated Breath-hold Examination
UTE: Ultrashort Echo Time
FRACTURE: Fast Field Echo Resembling a CT using Restricted Echo-spacing
PETRA: Pointwise Encoding Time Reduction with Radial Acquisition
SNR: Signal-to-Noise Ratio
MC: Mandibular Canal
TMJ: Temporomandibular Joint
TN: Trigeminal Nerve
LP: Lingual Plate
PTN: Peripheral Trigeminal Neuropathy
NVB: Neurovascular Bundle
MRN: Magnetic Resonance Neurography
ddMRI: Dental-Dedicated Magnetic Resonance Imaging
FFC-MRI: Fast Field Cycling Magnetic Resonance Imaging
